# Complete Genome Sequences of Three *Moraxella osloensis* Strains Isolated from Human Skin

**DOI:** 10.1128/genomeA.01509-17

**Published:** 2018-01-18

**Authors:** Jae Yun Lim, Ingyu Hwang, Munkhtsatsral Ganzorig, Shir-Ly Huang, Gyu-Sung Cho, Charles M. A. P. Franz, Kyoung Lee

**Affiliations:** a Department of Agricultural Biotechnology, Seoul National University, Seoul, South Korea; b Department of Bio Health Science, Changwon National University, Changwon, South Korea; c Institute of Microbiology and Immunology, National Yang-Ming University, Taipei, Taiwan; d Microbiology and Biotechnology of the Max Rubner-Institute, Federal Research Institute of Nutrition and Food, Kiel, Germany

## Abstract

Here, we present the complete whole-genome sequences of three *Moraxella osloensis* strains with octylphenol polyethoxylate-degrading abilities. These strains were isolated from human skin.

## GENOME ANNOUNCEMENT

*Moraxella osloensis*, a Gram-negative bacterium, is a part of the human skin microbiome (1). This strain is frequently involved in human infectious diseases (2). The complete genomes of the *M. osloensis* CCUG 350 and KMC41 strains, which were isolated from human cerebrospinal fluid and laundry, respectively, have been sequenced and published (3). However, the complete genome sequence of *M. osloensis* from human skin has not been previously reported.

Two *M. osloensis* strains, TT16 (KCTC 52863) and YHS (KCTC 52865), were isolated from the ear of a male college student, whereas the strain KSH (KCTC 52862) was isolated from the forehead of another male college student. The strains were isolated by swab sampling from the skin and direct streaking on minimal salts basal (MSB) medium containing 0.1% Triton X-100 (4). Ethical approval for subject sampling was granted by the Changwon National University ethics committee.

Total DNA of the cultured cells was extracted using the phenol extraction method (5). Whole-genome sequencing was performed on the RS II platform (PacBio) using 20-kb SMRTbell template libraries (National Instrumentation Center for Environmental Management [NICEM], Seoul National University). The obtained reads, with 220-, 230-, and 250-fold genome coverage of TT16, YHS, and KSH, respectively, were assembled *de novo* using Hierarchical Genome Assembly Process (HGAP) 3.0. Complete genome sequences were obtained by bioinformatics analysis, as previously described (6). Gene predictions and annotations were provided by the NCBI using the Prokaryotic Genome Annotation Pipeline (7). The SEED subsystem via the Rapid Annotations using Subsystems Technology (RAST) server was used for functional categorization of the predicted proteins (8).

The complete genomes of the three *M. osloensis* strains, TT16, YHS, and KSH, were 2,575,089, 2,575,090, and 2,483,272 bp in length, with 43.6%, 43.6%, and 43.9% G+C content, respectively. The respective strains had 2,376, 2,375, and 2,334 predicted coding genes. Strains TT16 and KSH had four plasmids, while strain YHS had three plasmids. All 3 genomes contained 47 tRNA genes, 4 noncoding RNA (ncRNA) genes, and 4 rRNA operons.

Xenobiotic degradation by the skin microbiome is a newly emerging research field (9). Alkylphenol and the short-ethoxylate-chain metabolites of octylphenol polyethoxylates (OPEs) formed by bacterial degradation reportedly have endocrine-disrupting activity (10, 11). Thus, the investigation of the catabolism of these compounds by the skin microbiome is warranted. The enzymes responsible for the transport and catabolism of OPEs have not been unequivocally identified in bacteria. Recently, involvement of the glyoxylate cycle in the catabolism of the ethoxy group formed by exoscission has been identified (12). The presence of isocitrate lyase (*aceA*) and malate synthetase (*glcB*) genes in the genomes of three *M. osloensis* strains indicated that the glyoxylate cycle is involved in the catabolism of OPEs. The whole-genome sequences of the *M. osloensis* strains can be used to determine the molecular bases for differential catabolism of OPEs by three different strains and the adaptation of *M. osloensis* to human skin.

### Accession number(s).

The complete genome sequences, including plasmids, of the *M. osloensis* TT16, YHS, and KSH strains were deposited in GenBank under the accession numbers CP024185 to CP024189, CP024176 to CP024179, and CP024180 to CP024184, respectively.
